# Repeated Administration of Cigarette Smoke Condensate Increases Glutamate Levels and Behavioral Sensitization

**DOI:** 10.3389/fnbeh.2018.00047

**Published:** 2018-03-16

**Authors:** In Soo Ryu, Jieun Kim, Su Yeon Seo, Ju Hwan Yang, Jeong Hwan Oh, Dong Kun Lee, Hyun-Wook Cho, Kyuhong Lee, Seong Shoon Yoon, Joung-Wook Seo, Insop Shim, Eun Sang Choe

**Affiliations:** ^1^Department of Biological Sciences, Pusan National University, Busan, South Korea; ^2^Research Center for Safety Pharmacology, Korea Institute of Toxicology, Daejeon, South Korea; ^3^Fundamental Research Division, Korea Institute of Oriental Medicine, Daejeon, South Korea; ^4^College of Fisheries Sciences, National Institute of Fisheries (NIFS), Busan, South Korea; ^5^Department of Physiology, Institute of Health Sciences, Gyeongsang National University School of Medicine, Jinju, South Korea; ^6^Department of Biology, Sunchon National University, Sunchon, South Korea; ^7^Inhalation Toxicology Research Center, Korea Institute of Toxicology, Jeongeup, South Korea; ^8^Department of Science in Korean Medicine, Kyung Hee University, Seoul, South Korea

**Keywords:** behavioral sensitization, biosensing, cigarette smoke condensate, glutamate, nicotine

## Abstract

Nicotine, a nicotinic acetylcholine receptor agonist, produces the reinforcing effects of tobacco dependence by potentiating dopaminergic and glutamatergic neurotransmission. Non-nicotine alkaloids in tobacco also contribute to dependence by activating the cholinergic system. However, glutamatergic neurotransmission in the dorsal striatum associated with behavioral changes in response to cigarette smoking has not been investigated. In this study, the authors investigated alterations in glutamate levels in the rat dorsal striatum related to behavioral alterations after repeated administration of cigarette smoke condensate (CSC) using the real-time glutamate biosensing and an open-field behavioral assessment. Repeated administration of CSC including 0.4 mg nicotine (1.0 mL/kg/day, subcutaneous) for 14 days significantly increased extracellular glutamate concentrations more than repeated nicotine administration. In parallel with the hyperactivation of glutamate levels, repeated administration of CSC-evoked prolonged hypersensitization of psychomotor activity, including locomotor and rearing activities. These findings suggest that the CSC-induced psychomotor activities are closely associated with the elevation of glutamate concentrations in the rat dorsal striatum.

## Introduction

Cigarette smoking is one of the greatest public health threats in the world. It is estimated that about 28 billion people, which is more than 40% of the global population are affected by tobacco smoke (World Health Organization, 2015). Cigarette smoke is a highly complex mixture of compounds that includes nicotine, non-nicotine alkaloids and over 4700 identified toxic compounds, such as, carcinogens and mutagens (Bhalla et al., 2009; Plöttner et al., 2012). Nicotine is regarded as a primary psychoactive alkaloid in tobacco because of its reinforcing effects on the initiation and maintenance of tobacco dependence (Benowitz, 2008). Stimulation of excitatory nicotinic acetylcholine receptors (nAChRs: α7 and α4β2 subtypes) after nicotine administration increases dopaminergic neurotransmission in the dorsal striatum, nucleus accumbens (NAc) and prefrontal cortex (PFC) by activating mesolimbic- and nigrostriatal projecting neurons from the ventral tegmental area (VTA) and substantia nigra pars compacta (SNpc), respectively (Mansvelder and McGehee, 2002; Zhao-Shea et al., 2011; Henley et al., 2013).

Stimulation of dopaminergic projections increases dopamine release, which is followed by glutamate release, in brain regions, such as, the dorsal striatum, NAc and PFC. This release has been shown to be associated with drug dependence (Kalivas, 2004; Zhao-Shea et al., 2011; Li et al., 2014). In addition, non-nicotine alkaloids in tobacco that are structurally similar to nicotine, such as, nornicotine, cotinine, anabasine and anatabine, activate dopaminergic and glutamatergic neurotransmission by stimulating nAChRs in the terminals of neurons (Huang and Hsieh, 2007; Maciuk et al., 2008; Clemens et al., 2009; Hoffman and Evans, 2013). Moreover, acetaldehyde, a constituent of tobacco, is also biologically active and plays role in reinforcing properties of tobacco smoking (Talhout et al., 2007; Brancato et al., 2014). These findings suggest that upregulation of the cholinergic system by non-nicotine compounds also plays a crucial role in tobacco dependence. However, the contribution made by these compounds to tobacco dependence is not well understood.

Glutamate is a key excitatory neurotransmitter in the mammalian brain and plays essential roles in nicotine dependence. For example, repeated exposure to nicotine increases glutamate release in the NAc, VTA and PFC by stimulating nAChRs (Lambe et al., 2003; Changeux, 2010; Shameem and Patel, 2012; Falasca et al., 2014). Repeated and challenge administrations of nicotine after nicotine abstinence following repeated nicotine exposure also potentiate glutamate levels in the dorsal striatum (Ryu et al., 2017). These findings suggest hyperactivation of the cholinergic system in mesolimbic and nigrostriatal projection neurons after nicotine exposure triggers the upregulation of glutamatergic neurotransmission.

Psychomotor sensitization, an addictive phenomenon, refers to hypersensitivity of motivated behaviors after repeated stimulation by drugs of abuse (Robinson and Berridge, 2008). This behavioral sensitization is mediated by signaling cascades linked to ionotropic and metabotropic glutamate receptors in the dorsal striatum, NAc and PFC (Kozell and Meshul, 2001; Kalivas, 2004; Kalivas et al., 2009; Lu et al., 2014; Liu and Steketee, 2016). Repeated or challenge exposure to nicotine increases psychomotor activity, including locomotor and rearing activities, by activating glutamatergic response in the dorsal striatum (Ryu et al., 2017). Together, these findings suggest that enhancement of glutamatergic neurotransmission in the dorsal striatum by nicotine exposure is closely associated with psychomotor sensitization caused by the psychoactive properties of nicotine. However, the effects of non-nicotine alkaloids on glutamate levels in the dorsal striatum are unclear. In this study, we sought to determine whether cigarette smoke regulates locomotor and rearing activities via altered glutamate levels in the dorsal striatum.

## Materials and Methods

### Animals

Adult male Sprague-Dawley rats weighing between 200 g and 230 g (6 weeks old) were purchased from Hyo-Chang Science Co. (Daegu, South Korea). A total of 30 rats were used in this study. Rats were separated into pairs and then allowed to acclimate to animal cages for a minimum of 5 days. Food and water were provided *ad libitum*. Rats were maintained on a 12-h light-dark cycle (light on at 8:00 AM) at a temperature and humidity of 21–23°C and 45%–55%, respectively, throughout the experimental period. All experimental measurements were performed in the light cycle. Experimental treatments were applied in a quiet room to minimize stress. All animal procedures were approved by the Institutional Animal Care and Use Committee of Pusan National University and conducted in accordance with the provisions of the National Institutes of Health Guide for the Care and Use of Laboratory Animals.

### Drugs

Nicotine hydrogen tartrate salt was purchased from Sigma-Aldrich (St. Louis, MO, USA), and then dissolved in 1% dimethyl sulfoxide (DMSO)/0.9% physiological saline (NaCl), and adjusted to pH 7.2–7.4 with sodium hydroxide (NaOH). This nicotine solution was freshly prepared immediately prior to each experiment. To investigate the psychoactive effects of cigarette smoke, cigarette smoke condensate (CSC) extracted from a 3R4F Kentucky reference cigarette (Sigma-Aldrich, St. Louis, MO, USA) was used in this study. The L-glutamic acid (Sigma-Aldrich) and L-ascorbic acid (Duchefa Biochemie B.V., Haarlem, Netherlands) were dissolved in phosphate buffered saline (PBS, pH 7.4) to make glutamate standard solutions and interfering analytical solutions, respectively, for *in vitro* calibration of glutamate biosensors. All working solutions of drug were always prepared immediately prior to every experiment.

### Preparation of CSC

CSC was prepared by the Research Center for Inhalation Toxicology at the Korea Institute of Toxicology (Jeongeup, South Korea) as previously described (Kwak and Lim, 2014; Kim et al., 2015). Cigarettes were conditioned for a minimum of 72 h prior to use at 21–23°C and 57%–63% relative humidity according to International Organization for Standardization (ISO) 3402 (International Organization of Standardization, 1999). CSC was generated using a 30 port smoking machine in accord with ISO 3308 (puff volume 35 ml, drawn over 2 s, time between puffs 60 s, and no vent blocking; International Organization of Standardization, 2012). All cigarettes were smoked to 3 mm beyond the end of the filter-tip paper according to ISO 4387 (International Organization of Standardization, 2000). CSC was prepared using a Cambridge filter pad (44 mm, Whatman, Maidstone, UK) after shaking for 30 min, such that the final concentration of total particulate matter (TPM) was 20 mg/mL in methanol. This methanol was subsequently vaporized in a vacuum dry oven for 1 day, after which dried CSC was dissolved in 1% DMSO/0.9% NaCl. Dissolved CSC samples were passed through 0.45 μm PTFE sterile filters and stored at −80°C until used. The nicotine and minor alkaloid contents of the CSC are presented in Table 1.

**Table 1:** Contents of nicotine and minor alkaloids in cigarette smoke condensate (CSC; μg/mL).

Types	Description	Nicotine	(R,S)-Nornicotine	(R,S)-Anabasine	(R,S)-Anatabine
3R4F	Reference cigarette	4069.3	25.29	412.60	6.06

### Administration of Nicotine and CSC

The dose used for subcutaneous (s.c.) nicotine (0.4 mg/kg/day) administration was determined from previous studies (Matta et al., 2007; Ryu et al., 2017). The extracted CSC was dissolved in 1% DMSO/0.9% NaCl and diluted to make working solutions with a nicotine content of 0.4 mg, and were then adjusted to pH 7.2–7.4 with NaOH. Animals were subcutaneously administered saline, nicotine or CSC (1.0 mL/kg/day) for 14 consecutive days. Rats were divided into three different groups in each experiment as follows: (1) 14 days repeated saline group (*n* = 5); (2) 14 days repeated nicotine group (*n* = 5); and (3) 14 days repeated CSC group (*n* = 5).

### Surgery for Real-Time Glutamate Biosensing

Surgery for glutamate biosensing was performed as previously described (Ryu et al., 2017). Briefly, rats were anesthetized with a mixture of Zoletil 50 (tiletamine; 18.75 mg/kg; Virbac Korea, Seoul) and Rompun (xylazine; 5.8 mg/kg; Bayer Korea, Seoul) by intraperitoneal (i.p.) injection, and then fixed in a stereotaxic apparatus. Under aseptic conditions, a BASi Rat Guide Cannula (Part #7030, Pinnacle Technology, Lawrence, KS, USA; inner diameter: 0.7 mm, 10 mm in length) was surgically implanted into the center of the right dorsal striatum (1.0 mm anterior to the Bregma, 2.5 mm right of the midline, and 5 mm below the surface of the skull) to allow the insertion of a glutamate biosensor (Supplementary Figure S1A). BLE Rat Hat Bottoms (Part #8108, Pinnacle Technology) were positioned on the hat of the rat to enable potentiostat placement (Part #8172, No. 9225/9226, Pinnacle Technology), after which BLE Rat Hat Bottoms were covered with BLE Rat Hat Tops (Part #8107, Pinnacle Technology) until glutamate biosensing (Supplementary Figure S1A). Following surgery, rats were given a minimum of 5 days of recovery in animal cages, and then treated with 0.1 mL of gentamycin (i.p., Eagle Vet, Seoul, South Korea) prior to the first administration of saline, nicotine or CSC. After glutamate biosensing, the physical accuracy of guide cannula implantation of 15 rats was verified by reconstruction of guide cannula placements (Supplementary Figure S1B). The possibility of gliosis caused by the guide cannula implantation and glutamate biosensor insertion was verified by Nissl staining (data not shown).

### *In Vitro* Calibration and *in Vivo* Real-Time Glutamate Biosensing

Commercial L-glutamate oxidase-based glutamate biosensors (glutamate biosensors; Part #7002, Pinnacle Technology) and L-glutamate oxidase-free glutamate biosensors (glutamate null biosensors; Pinnacle Technology) were used in this study. A working model of the glutamate biosensor in the dorsal striatum is shown in Supplementary Figure S2 (modified from the previous study, Naylor et al., 2011). Briefly, a platinum-iridium (Pt-Ir) wire (diameter 0.18 mm) with a 1.0 mm long sensing tip and an Ag/AgCl reference electrode were incorporated with the active electrode. On the active surface of glutamate biosensors, glutamate oxidase converts glutamate to α-ketoglutarate and H_2_O_2_, and the H_2_O_2_ produced then diffuses through the selective membrane to the Pt-Ir surface, where it is detected as an amperometric oxidation current generated by an applied potential of +0.6 V^26^. Before and after calibrations were conducted *in vitro* in PBS (pH 7.4) by gradually increasing the concentrations of glutamate from 0 to 1, 2, 3 and 4 μM. A single addition of 250 μM ascorbic acid, which commonly causes biological interference, did not interfere with glutamate detection (data not shown), which is consistent with the results of previous studies on glutamate biosensors (Naylor et al., 2011; Lenoir and Kiyatkin, 2013). All analytical solutions were freshly prepared before and after calibrations. Because the sensitivity of the biosensor for glutamate detection is directly influenced by temperature (Wakabayashi and Kiyatkin, 2012), all calibration procedures were performed at 37°C and a minimum of 5 min was allowed prior to measurements to ensure conditions had stabilized. In our previous study, since acute and repeated nicotine administration did not alter temperature in the dorsal striatum in freely moving rats (Ryu et al., 2017), we excluded a possibility of influence on glutamate biosensor *in vivo* in this study. In addition, since glutamate biosensor outputs are inversely related to changes in current *in vivo*, rats were acclimated to the testing environment for a minimum of 120 min after biosensor insertion until current changes had stabilized. When changes in currents reached a relatively stabilized baseline, real-time glutamate biosensing in the dorsal striatum of freely moving rats was conducted for 60 min after the final administrations of repeated saline, nicotine, or CSC in home cage. Since the baseline current of individual rats was slightly influenced by each glutamate biosensor, the absolute values of the current caused by saline or nicotine administration were transformed into the relative values of the current by normalizing the basal value to be 0 nA. These changes in the current of the dorsal striatum were then converted into changes in the concentrations of glutamate based on the sensitivity of each glutamate biosensor adjusted by its after-calibration plots. Data were sampled at 1 Hz using SIRENIA acquisition software (version 1.6.1, Pinnacle Technology).

### Behavioral Assessments

Behavioral assessments were performed as previously described (Oh et al., 2015). Briefly under illuminated and sound-attenuated conditions, locomotor activity (total distance traveled as determined by horizontal beam breaks) and rearing activity for stereotypy movement (counts were determined by vertical beam breaks) in an open-field using an infrared photocell-based, automated Opto-Varimax-4 Auto-Track (Columbus Instruments, Columbus, OH, USA) after saline, nicotine, or CSC administration for 14 days. Rats were acclimated to a behavioral test chamber (width: 44.5 cm, length: 44.5 cm, height: 24 cm) for a minimum of 6 days to avoid environmental variations prior to experiments. Three pairs of sensors were positioned on x-, y- (horizontal), and z- (vertical, placed above normal animal height) axes to provide coordinates that recognized locomotor activity and rearing. Each sensor pair produced 16 infrared light beams that intersected the animal cage (beam scan rate = 10 Hz). The Auto-Track system senses the presence of animals by receiving infrared beam interruptions. Locomotor and rearing activities were recorded in 1 min intervals for 30 and 60 min before and after drug administration, and data were transferred from all sensors to a computer using Opto-Varimex 4 Auto Track Rapid Release software (v4.99B software, Columbus Instruments).

### Statistics

For statistical analysis, detected currents by the glutamate biosensors were converted into glutamate concentrations. Alterations in locomotor and rearing activities were recorded as total distance traveled (cm) and rearing (count) in 1 min intervals for 60 min. Statistical significances between groups were determined using the two-tailed unpaired *t*-test, and one- or two-way analysis of variance (ANOVA) with repeated measures (RM), followed by Tukey’s or Bonferroni’s *post hoc* test, respectively. Analysis was conducted using GraphPad Prism 6 (GraphPad Software, La Jolla, CA, USA). Data were presented as mean ± SEM for each group (*n* = 5 per group). Statistical significance was accepted for *p* values < 0.05.

## Results

### Real-Time Glutamate Biosensing in the Rat Dorsal Striatum

**Figure 1:**
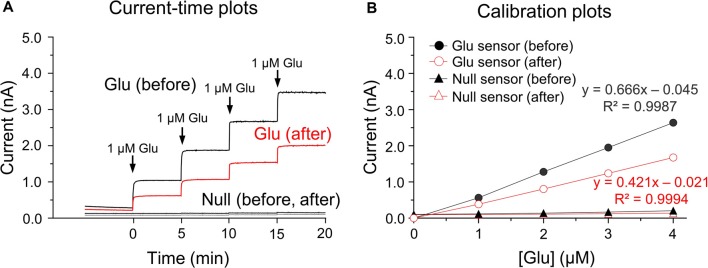
*In vitro* current-time and calibration plots. Current (I)-time plots obtained after the successive additions of glutamate standard solution in phosphate buffered saline (PBS) at pH 7.4 before and after biosensing **(A)**. Calibration plots of currents-glutamate concentrations [Glu] detected by the glutamate biosensors and glutamate null sensors used for *in vitro* calibration **(B)**. Note no changes in currents or glutamate concentrations in response to the addition of glutamate standard solution for glutamate null sensors.

Glutamate microbiosensors were calibrated and control experiments were conducted before and after biosensing glutamate concentrations in the rat dorsal striatum. In the current-time plots, the sensitivity of glutamate biosensors before measurements was 0.797 ± 0.015 nA/μM, and an approximate two-fold decrease (0.454 ± 0.014 nA/μM) in glutamate sensitivity was observed (Figure 1A). Linear calibration plots were obtained using steady-state currents and glutamate concentrations in the range 0–4 μM (Figure 1B). A single addition of ascorbic acid (250 μM) did not interfere with glutamate detection before and after calibrations (data not shown). *In vitro* calibration, there were no changes in currents and glutamate concentrations in response to the addition of glutamate standard solutions within the glutamate null sensors (before biosensing: 0.012 ± 0.011 nA/μM; after biosensing: 0.007 ± 0.002 nA/μM; Figure 1B). For this reason, the present study was conducted with glutamate biosensors only for the following experiments.

### Repeated Administration of CSC Significantly Increased Extracellular Glutamate Concentrations in the Rat Dorsal Striatum

**Figure 2:**
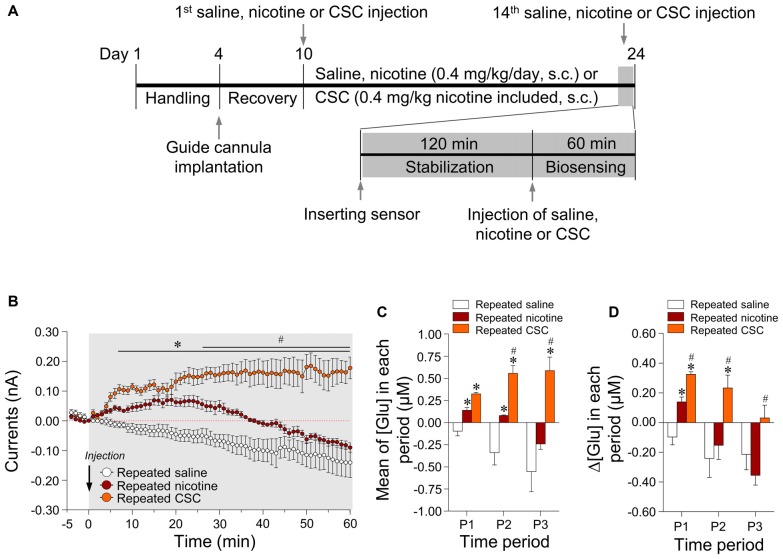
Changes in output currents and glutamate concentrations in the dorsal striatum after repeated administration of nicotine and cigarette smoke condensate (CSC). Timeline of real-time glutamate biosensing in the dorsal striatum **(A)**. Changes in currents **(B)** were converted to glutamate concentrations [Glu] **(C)** and rates of change in glutamate concentrations (Δ[Glu]) **(D)** after the repeated administration of saline, nicotine or CSC in the dorsal striatum during the three periods. P1, 0–20 min; P2, 20–40 min; P3, 40–60 min. *p < 0.05 vs. repeated saline group; ^#^*p* < 0.05 vs. repeated nicotine group.

Since 14 days of repeated nicotine administration increases glutamate levels (Ryu et al., 2017), we investigated whether 14 days of repeated exposure to CSC alters extracellular glutamate concentrations in the rat dorsal striatum. The timeline for repeated treatment with saline, nicotine, or CSC and the real-time biosensing of extracellular glutamate release are presented in Figure 2A. Repeated saline and nicotine-treated groups were used as negative and positive controls, respectively, for the CSC in all subsequent experiments. Repeated nicotine administration significantly increased currents (Time: *F*_(60,240)_ = 8.734, *p* < 0.0001; Treatment: *F*_(1,4)_ = 21.87, *p* = 0.0185; Time × Treatment: *F*_(60,240)_ = 2.320, *p* < 0.0001) as compared with the repeated saline control group (Figure 2B). Currents changes caused by repeated nicotine administration increased up to 20 min time point, and then decreased to baseline at 30–40 min and remained at baseline levels until the end of recording. Thus, we analyzed the changes in glutamate concentrations dividing with three different time period as follows: P1, 0–20 min; P2, 20–40 min; P3, 40–60 min. The results demonstrated repeated nicotine administration significantly increased mean glutamate concentrations at P1 (*t*_(8)_ = 3.775, *p* = 0.0054) and P2 (*t*_(8)_ = 3.014, *p* = 0.0167), but not P3 (Figure 2C). Like mean glutamate concentrations, rates of changes in glutamate concentrations (Δ[Glu]) after repeated nicotine administration were significantly increased at P1 (*t*_(8)_ = 3.775, *p* = 0.0054), but not at P2 or P3 as compared with the repeated saline control group (Figure 2D).

In the CSC group, repeated exposure significantly increased currents as compared with the repeated saline group (Time: *F*_(60,240)_ = 0.4884, *p* = 0.9993; Treatment: *F*_(1,4)_ = 134.4, *p* = 0.0003; Time × Treatment: *F*_(60,240)_ = 12.66, *p* < 0.0001) and the repeated nicotine group (Time: *F*_(60,240)_ = 4.666, *p* < 0.0001; Treatment: *F*_(1,4)_ = 11.46, *p* = 0.0277; Time × Treatment: *F*_(60,240)_ = 6.006, *p* < 0.0001; Figure 2B). Repeated administration of CSC also significantly increased mean glutamate concentrations at P1–P3 and at P2–P3 (P1: *F*_(2,12)_ = 31.88, *p* < 0.0001; P2: *F*_(2,12)_ = 22.55, *p* < 0.0001; P3: *F*_(2,12)_ = 13.38, *p* = 0.0009) as compared with the repeated saline and nicotine control groups, respectively (Figure 2C). The rates of changes in glutamate concentrations after repeated CSC administration was also significantly increased at P1–P2 and at P1–P3 (P1: *F*_(2,12)_ = 31.88, *p* < 0.0001; P2: *F*_(2,12)_ = 5.879, *p* = 0.0166; P3: *F*_(2,12)_ = 5.130, *p* = 0.0245) as compared with the repeated saline and nicotine control groups, respectively (Figure 2D). Absolute values of rates of changes in glutamate concentrations in the dorsal striatum during the three time periods after the repeated administrations of saline, nicotine, or CSC are provided in Table 2.

**Table 2:** The rates of changes in the glutamate concentrations of the dorsal striatum obtained by real-time glutamate biosensing during three periods after the repeated administration of saline, nicotine, or CSC.

Groups	Δ[Glu] (nM)		
	P1	P2	P3
14 days repeated saline	−97.01 ± 52.86	−242.20 ± 126.7	−214.30 ± 103.00
14 days repeated nicotine	138.60 ± 33.18*	−152.70 ± 94.73	−355.00 ± 65.29
14 days repeated CSC	324.40 ± 17.38*	232.50 ± 86.19*,^#^	30.76 ± 86.06*,^#^

P1, 0–20 min; P2, 20–40 min; P3, 40–60 min. *,^#^Represent an increase in the rates of change of extracellular glutamate concentrations as compared with the repeated saline and nicotine control groups, respectively.

### Repeated Administration of CSC Significantly Increased Locomotor and Rearing Activities

**Figure 3:**
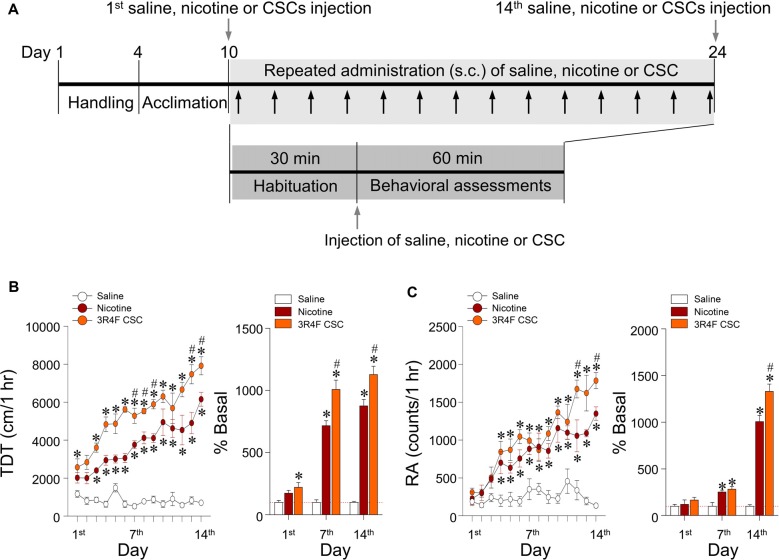
Changes in behavioral activities after repeated administration of nicotine and CSC. Timeline showing the results of behavioral assessments of locomotor and rearing activities** (A)**. Arrows in gray shadow represent the measurements of locomotor and rearing activities once a day for 14 days. Changes in locomotor activity expressed as total distance traveled (TDT; **B**) and rearing activity (RA; **C**) following repeated administrations of saline, nicotine or CSC. *p < 0.05 vs. repeated saline group; ^#^*p* < 0.05 vs. repeated nicotine group.

This study was conducted to measure the psychoactive effects of CSC on psychomotor sensitization based on measurements of locomotor and rearing activities over 60 min after repeated administration of CSC (Figure 3A). Repeated nicotine administration significantly increased locomotor activity (Time: *F*_(13,52)_ = 16.72, *p* < 0.0001; Treatment: *F*_(2,8)_ = 437.2, *p* < 0.0001; Time × Treatment: *F*_(26,104)_ = 8.494, *p* < 0.0001; 1st day: *F*_(2,12)_ = 4.959, *p* = 0.0269; 7th day: *F*_(2,12)_ = 84.29, *p* < 0.0001; 14th day: *F*_(2,12)_ = 116.8, *p* < 0.0001; Figure 3B) and RA (Time: *F*_(13,52)_ = 19.03, *p* < 0.0001; Treatment: *F*_(2,8)_ = 42.66, *p* < 0.0001; Time × Treatment: *F*_(26,104)_ = 5.199, *p* < 0.0001; 1st day: *F*_(2,12)_ = 0.9499, *p* = 0.4140; 7th day: *F*_(2,12)_ = 10.29, *p* = 0.0025; 14th day: *F*_(2,12)_ = 110.7, *p* < 0.0001) as compared with the saline control group (Figure 3C). These results demonstrated repeated administration of CSC significantly increased locomotor activity (Time: *F*_(13,52)_ = 19.03, *p* < 0.0001; Treatment: *F*_(2,8)_ = 42.66, *p* < 0.0001; Time × Treatment: *F*_(26,104)_ = 5.199, *p* < 0.0001; (1st day: *F*_(2,12)_ = 0.9499, *p* = 0.4140; 7th day: *F*_(2,12)_ = 10.29, *p* = 0.0025; 14th day: *F*_(2,12)_ = 110.7, *p* < 0.0001; Figure 3B) and RA (Time: *F*_(13,52)_ = 19.03, *p* < 0.0001; Treatment: *F*_(2,8)_ = 42.66, *p* < 0.0001; Time × Treatment: *F*_(26,104)_ = 5.199, *p* < 0.0001; 1st day: *F*_(2,12)_ = 0.9499, *p* = 0.4140; 7th day: *F*_(2,12)_ = 10.29, *p* = 0.0025; 14th day: *F*_(2,12)_ = 110.7, *p* < 0.0001) as compared with the repeated saline and nicotine control groups (Figure 3C).

**Figure 4:**
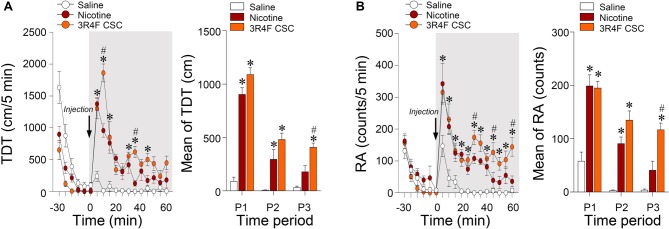
Changes in locomotor and rearing activities after repeated administration of nicotine and CSC. Changes in TDT **(A)** and RA **(B)** after the 14 days repeated administration of saline, nicotine or CSC during the three periods. P1, 0–20 min; P2, 20–40 min; P3, 40–60 min. *p < 0.05 vs. 14 days repeated saline group; ^#^*p* < 0.05 vs. 14 days repeated nicotine group.

Since hypersensitive glutamate levels by repeated administration of CSC may be associated with psychomotor sensitization, we analyzed behavioral changes at the three periods. Repeated administration of CSC significantly increased both locomotor activity (Time: *F*_(12,48)_ = 32.46, *p* < 0.0001; Treatment: *F*_(2,8)_ = 51.01, *p* < 0.0001; Time × Treatment: *F*_(24,96)_ = 10.66, *p* < 0.0001; P1: *F*_(2,12)_ = 89.33, *p* < 0.0001; P2: *F*_(2,12)_ = 14.24, *p* = 0.0007; P3: *F*_(2,12)_ = 20.06, *p* = 0.0001; Figure 4A) and RA (Treatment: *F*_(2,8)_ = 39.44, *p* < 0.0001; Time: *F*_(12,48)_ = 18.82, *p* < 0.0001; Interaction: *F*_(24,96)_ = 3.513, *p* < 0.0001; P1: *F*_(2,12)_ = 21.87, *p* < 0.0001; P2: *F*_(2,12)_ = 29.71, *p* = 0.0007; P3: *F*_(2,12)_ = 21.38, *p* = 0.0001; Figure 4B). Changes in locomotor and rearing activities in each time period following the repeated administration of saline, nicotine or CSC are listed in Table 3.

**Table 3:** Changes in locomotor and rearing activities during the three periods after the repeated administration of saline, nicotine, or CSC.

Groups	Locomotor activity		
	P1	P2	P3
14 days repeated saline	89.35 ± 39.49	7.25 ± 2.78	30.60 ± 14.53
14 days repeated nicotine	904.30 ± 62.05*	295.40 ± 92.53*	178.30 ± 59.50*
14 days repeated CSC	1090.00 ± 64.03*	481.80 ± 58.98*	407.90 ± 40.69*,^#^
		**Rearing activity**
14 days repeated saline	57.85 ± 17.10	2.75 ± 1.20	3.70 ± 1.91
14 days repeated nicotine	198.80 ± 20.87*	90.60 ± 12.62*	40.75 ± 17.19*
14 days repeated CSC	195.00 ± 12.49*	134.80 ± 17.18*	116.60 ± 12.87*,^#^

P1, 0–20 min; P2, 20–40 min; P3, 40–60 min. *,^#^Represent an increase in locomotor and rearing activities as compared with the repeated saline and nicotine control groups, respectively.

### Changes in Extracellular Glutamate Concentrations in the Rat Dorsal Striatum Were Correlated With Locomotor and Rearing Activities After Repeated CSC Administration

**Figure 5:**
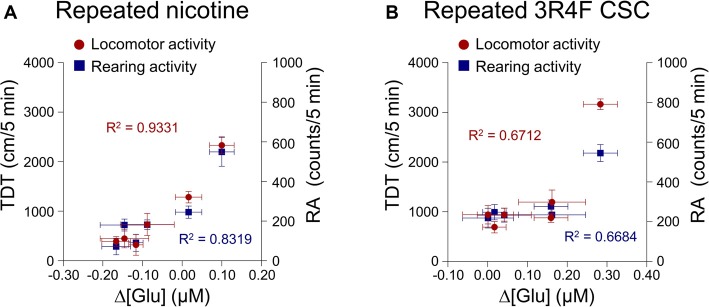
Correlation coefficients for the relationships between the rates of changes in glutamate concentrations of the dorsal striatum and behavioral changes. Increases in the rates of changes in glutamate concentrations after repeated nicotine **(A)** or CSC administration **(B)** have high correlation coefficients with repeated nicotine or CSC-induced increases in locomotor and rearing activities. Left and right *y*-axes indicate the real values of TDT and RA, respectively.

This study was conducted to explore the correlation between changes in extracellular glutamate concentrations in the rat dorsal striatum and behavioral changes after the repeated administration of nicotine or CSC. The analysis was performed using Pearson’s correlation. The results showed high correlation coefficients for relations between changes in extracellular glutamate concentrations and behavioral changes of locomotor activity (*R*^2^ = 0.9331, *p* = 0.0017) and RA (*R*^2^ = 0.8319, *p* = 0.0113) after repeated nicotine exposure (Figure 5A). Changes in glutamate concentrations after repeated CSC administration were also highly correlated with locomotor activity (*R*^2^ = 0.6712, *p* = 0.0416) and RA (*R*^2^ = 0.6684, *p* = 0.0469; Figure 5B).

## Discussion

The dorsal striatum is a forebrain structure that integrates nigrostriatal dopaminergic projections from the SNpc and glutamatergic projections from the somatosensory cortices, which are closely associated with drug-mediated motivation (Yager et al., 2015). Previous studies have demonstrated cigarette smoke upregulates the functions and expressions of dopamine active transporters in the dorsal striatum and NAc (Danielson et al., 2014). Exposure to acetaldehyde, a component of cigarette smoke, increases the firing rate and burst firing of the VTA neurons, which leads to dopamine release in a dose-dependent manner in rats (Foddai et al., 2004; Bhalla et al., 2009; Plöttner et al., 2012). Similarly, the present study shows that repeated administration of CSC significantly increased extracellular glutamate concentrations in the rat dorsal striatum. Together, these findings suggest that cigarette smoke containing addictive molecules triggers the upregulation of glutamatergic neurotransmission in the rat dorsal striatum.

Non-nicotine compounds, such as anabasine, cotinine and nornicotine, induce an increase in midbrain dopamine release by stimulating nAChRs in a calcium-dependent manner (Dwoskin et al., 1993; O’Leary et al., 2008). Furthermore, it has been reported that cigarette smoke upregulated the expression of nAChRs and the binding properties of nAChRs more than nicotine treatment alone in a human neuroblastoma cell line (Ambrose et al., 2007). A previous study demonstrated that CSC including non-nicotine compounds had relatively longer half-life in terms of its stimulus effects as compared with nicotine alone (Lee et al., 2016). Consistent with these findings, we found repeated CSC administration increased extracellular glutamate concentrations in the dorsal striatum and prolonged its effects on glutamate levels as compared with repeated nicotine administration alone. Taken together, these findings suggest that the longer effects of non-nicotine components in tobacco products act synergistically with nicotine to stimulate nAChRs and potentiate glutamate levels in the dorsal striatum.

Glutamate plays a crucial role in the mediation of drug-induced synaptic plasticity in drug addiction (Kalivas et al., 2009). Psychomotor sensitization has been shown to be associated with glutamatergic neurotransmission in the ventral midbrain (Vezina, 2004; Steketee and Kalivas, 2011; Lu et al., 2014). Repeated intravenous injections of nicotine increased glutamate levels in the NAc and VTA of rat and locomotion (Lenoir and Kiyatkin, 2013). A previous study conducted by our laboratory demonstrated that nicotine challenge followed by nicotine withdrawal increased behavioral changes and potentiated glutamate levels in a α7 nAChR associated manner in the dorsal striatum (Ryu et al., 2017). Consistent with these findings, in the present study showed that sensitized psychomotor activities (locomotor and rearing activities) following repeated CSC administration were significantly correlated with increase in extracellular glutamate concentrations in the dorsal striatum. These findings suggest that repeated CSC-induced hyperactivity in glutamate levels causes sensitization of behaviors.

In a previous study, repeated intravenous administration of a cocktail of nicotine and five minor alkaloids (anabasine, nornicotine, anatabine, cotinine and myosmine) was found to enhance nicotine-induced psychomotor sensitization (Clemens et al., 2009). In an intracranial self-stimulation study, alkaloids in tobacco appeared to mimic the addiction-related effects of nicotine in rats (Harris et al., 2015). Previous studies reported that acetaldehyde administration produces a conditioned place preference in rodents and interacts with nicotine to increase responding in a stringent self-administration acquisition test (Belluzzi et al., 2005; Spina et al., 2010). However, the systemic administration of cotinine alone did not affect sensitization of locomotor activity in rats (Wiley et al., 2015; Ryu et al., 2017). Similarly, in the present study repeated exposure to CSC induced hypersensitization of psychomotor activity and sustained its effects for longer than repeated nicotine administration alone. Taken together, these findings suggest that non-nicotine constituents in cigarette smoke synergistically upregulate the psychoactive effects of nicotine and contribute to the potentiation of psychomotor behaviors. This conclusion may be limited to a few non-nicotinic minor alkaloids in cigarette smoke components. Considering cigarette smoke also contains psychoactive components, such as acetaldehyde, and many other agents, analyses of every cigarette smoke component are needed in future studies (Talhout et al., 2007).

In summary, repeated administration of CSC in freely moving rats increased extracellular glutamate concentrations in the rat dorsal striatum more than repeated nicotine exposure. Furthermore, our data suggest this long-lasting increase in glutamate levels in the dorsal striatum may lead to the hypersensitization of psychomotor behaviors. These findings suggest that the hyperactivation of glutamatergic response in the dorsal striatum via the synergistic actions of nicotine and non-nicotine alkaloids after repeated exposure to cigarette smoke is a neurochemical event, leading to behavioral changes in locomotor and rearing activities. Integrative analysis of neurochemical actions of nicotine and non-nicotine components contained in CSC contributing to glutamate response-mediated behavioral sensitization remains to be determined in future studies.

## Author Contributions

ISR and ESC designed the research. ISR, JK, SYS, JHY and KL conducted the research. ISR, DKL, JHO, H-WC, IS, SSY, J-WS and ESC analyzed the data. ISR and ESC wrote the manuscript.

## Conflict of Interest Statement

The authors declare that the research was conducted in the absence of any commercial or financial relationships that could be construed as a potential conflict of interest.
