# Supplementary material for: Repeated Administration of Cigarette Smoke Condensate Increases Glutamate Levels and Behavioral Sensitization
Source: Front Behav Neurosci. 2018 Mar 16;12:47. doi: 10.3389/fnbeh.2018.00047 (PMC5864865; doi:10.3389/fnbeh.2018.00047)
Supplement: Supplementary file 1 [file Image_1.pdf]

Supplementary Material

Repeated administration of cigarette smoke condensate increases glutamate levels and behavioral sensitization

In Soo Ryu PhD[1,6], Jieun Kim BSc[1], Su Yeon Seo PhD[1,8], Ju Hwan Yang PhD[1], Jeong Hwan Oh PhD[2], Dong Kun Lee PhD[3], Hyun-Wook Cho PhD[4], Kyuhong Lee PhD[5], Seong Shoon Yoon PhD[6], Joung-Wook Seo PhD[6], Insop Shim PhD[7], Eun Sang Choe PhD[1,*]

*** Correspondence:** Eun Sang Choe, eschoe@pusan.ac.kr

Supplementary Figure S1. Ryu et al.

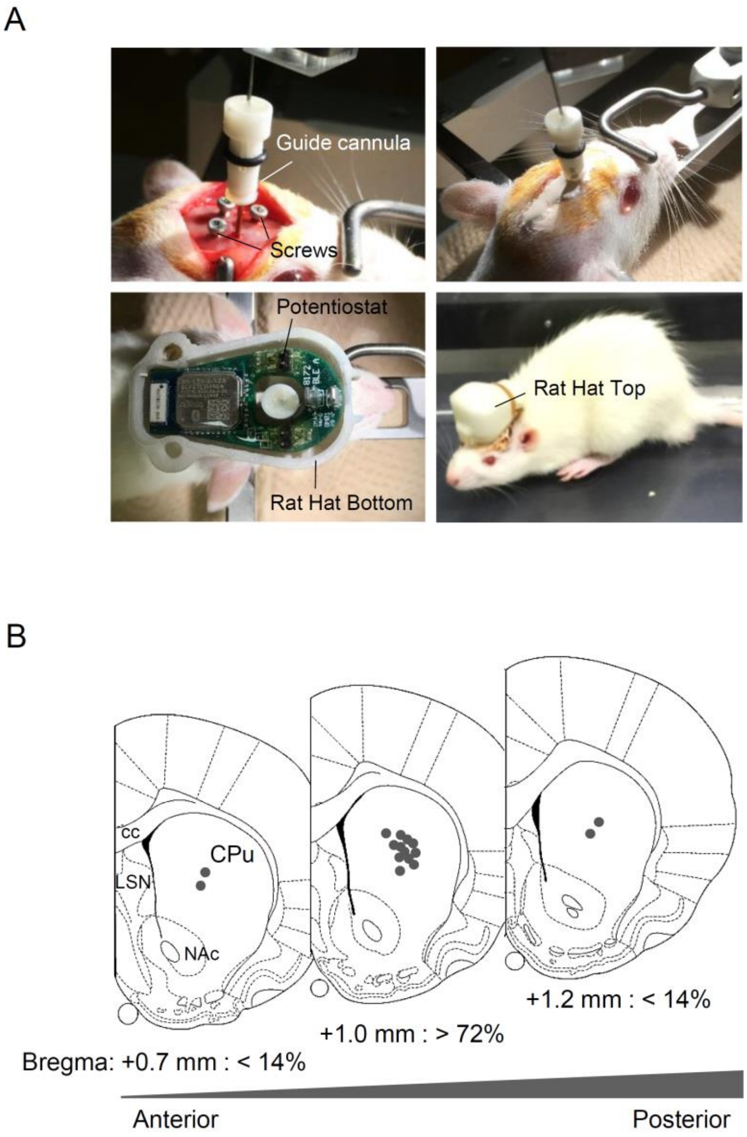

Supplementary Figure S1. Surgery for glutamate biosensing in the dorsal striatum (A). A guide cannula was implanted into the center of the right dorsal striatum (CPu) (coordinates: 1.0 mm anterior to the bregma, 2.5 mm right of the midline, and 5.0 mm below the surface of the skull) and then fixed with dental cement. A potentiostat was placed on the Rat Hat Bottom and covered with the Rat Hat Top. Brain sections showing the placements of glutamate biosensors in the right dorsal striatum. Percentage (%) of each brain section represents the accuracy of guide cannula implantation of 15 rats. (B). cc, corpus callosum; LSN, lateral septal nuclei; NAc, nucleus accumbens.

Supplementary Figure S2. Ryu et al.

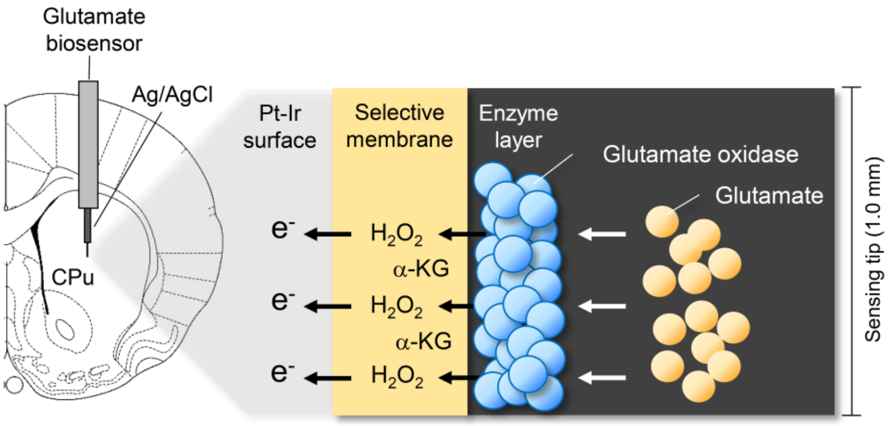

Supplementary Figure S2. Schematic diagram illustrating the glutamate biosensor and the working model used for the detection of extracellular glutamate. α-KG, alpha-ketoglutarate; Pt-Ir, platinum-iridium.
